# Enhancing Ultrasonic Echo Response of AlN Thin Film Transducer Deposited by RF Magnetron Sputtering

**DOI:** 10.3390/s24175820

**Published:** 2024-09-07

**Authors:** Fengqi Wang, Qinyan Ye, Kun Luo, Xulin He, Xiaolong Ran, Xingping Zheng, Cheng Liao

**Affiliations:** Chengdu Development Center of Science and Technology of CAEP, Chengdu 610299, China; wangle_17@126.com (F.W.);

**Keywords:** pretightening stress, AlN thin film, transducer, ultrasonic echo, magnetron sputtering, structure optimization

## Abstract

Accurate measurement of the pretightening stress for bolts has great significance for improving the assembly quality and safety, especially in severe environments. In this study, AlN thin film transducers were deposited on GH4169 nickel base alloy bolts using the RF magnetron sputtering, enabling a systematic investigation into the correlation between structures and the intensity of ultrasonic echo signals. Employing the finite element method resulted in consistency with the experimental data, enabling further exploration of the enhancement mechanism. With the increasing thickness of both the piezoelectric layer and the electrode layer, the intensity of the ultrasonic echo signals saw a great enhancement. The maximum-intensity observed increase is 14.7 times greater than that of the thinnest layers. Specifically, the thicker piezoelectric layer improves its mechanical displacement, while the increased thickness of the electrode layer contributes to better densification. An electrode diameter of nearly 4 mm is optimal for an AlN thin film transducer of M8 bolts. For pretightening the stress measurement, the sample with a strong and stable echo signal shows a low measurement error of pretightening below ±2.50%.

## 1. Introduction

In severe environments, the safety of the connection structures is a key factor in the reliable operation of important equipment, such as aero-engines [1], high-speed trains [2], nuclear reactors [3], and various other industries. Bolts play an important role in component connections and pressure sealing. Monitoring the pretightening stress of bolts is essential for maintaining the safety and reliability of connected structures. Among various preload measurement techniques, ultrasonic measurement techniques based on an acoustoelastic effect have received much attention, with the advantages of real-time, rapid, and accurate measurement [4,5,6]. In this method, an ultrasonic signal is transmitted from the top to the bottom of the bolt by the ultrasonic transducer and then reflected back. By recording the time difference between the signals from unstressed and stressed bolts, the pretightening stress within the bolt can be determined [7,8]. Obviously, the stability and intensity of the received ultrasonic signal are the key factors for measuring the pretightening stress in bolts.

Up to now, the ultrasonic sensor for pretightening stress mainly contains a piezoelectric probe [9], piezoelectric ceramics [7], and a permanently mounted transducer system (PMTS) [10]. The piezoelectric probe and piezoelectric ceramic methods, mainly using PZT ceramics, have stable and strong ultrasonic echo signals. However, both the viscous couplant layer and the PZT materials are not suitable for use at elevated temperatures over an extended period. The PMTS technique directly deposits the transducer on the head of the bolt through physical vapor deposition (PVD) [10,11], which can avoid the viscous couplant layer. Zeng et al. reported LiNiO_3_ thin film transducers deposited on Inconel bolts by RF magnetron sputtering, and these transducers exhibit a good ultrasonic echo response at high temperatures [12,13]. The piezoelectric oxide is difficult to deposit and may have impurities in the phase [14]. Among the piezoelectric candidates, wurtzite aluminum nitride (*w*-AlN) can withstand extreme harsh environments because of its high sound velocity, high-quality factor, strong chemical stability, high temperature resistance, etc. [15,16,17]. AlN demonstrates stable piezoelectric properties even at elevated temperatures of up to 1000 °C in real time [18], a characteristic that makes it highly valuable for applications in extreme environments where conventional piezoelectric materials may fail or degrade. It is well known that the *w*-AlN films with a *c*-axis crystal orientation exhibit superior piezoelectric properties. To obtain a pure AlN film with a (0002) preferential orientation, RF magnetron sputtering has been widely employed in many studies [19,20,21]. However, its small piezoelectric coefficient (*d*_33_ of 5.5 pC/N [15]) hinders its ability on transmitting and receiving the ultrasonic signals of the transducer. Through the use of doping, many studies have successfully increased the *d*_33_ value, employing both experiments [19,22,23,24] and calculations [25,26,27,28]. By optimizing the geometric structure of the transducer, Wu et al. reported an optimal PZT transducer structure with higher electromechanical coupling coefficients [29]. Zeng et al. enhanced the ultrasonic echo response of LiNiO_3_ thin film transducer for bolts by increasing the thickness of LiNiO_3_ piezoelectric films [13]. But there is little work on the AlN thin-film transducers, which are grown in situ on top of the bolts for the purpose of measuring the pretightening stress.

This study aimed to investigate the characteristics of ultrasonic echo signals from AlN thin-film transducers under varying structural parameters, such as the thickness of the piezoelectric layer, the thickness of the electrode layer, and the diameter of the electrode. The validity of the theoretical models proposed in this paper has been verified through experimentation, which allows for further exploration of the enhancement mechanism of ultrasonic echo signals. On this basis, the pretightening stress is measured by an AlN thin film transducer, resulting in a lower error rate.

## 2. Experimental Details

### 2.1. Transducer Integration

**Figure 1:**
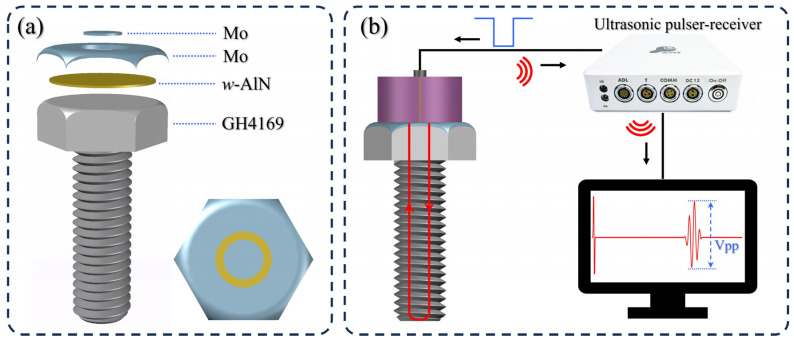
The structure diagram of AlN thin film ultrasonic transducer (**a**) and the experimental device for ultrasonic response measurement (**b**).

AlN thin film transducers were deposited on polished GH4169 nickel base alloy bolts (M8 × 30 mm) and Si(100) using RF magnetron sputtering. An Al target and Mo target were prepared using smelting technology (99.999% purity, Hangzhou Kaiyada semiconductor Material Co., Ltd., Hangzhou, China). Before deposition, the GH4169 bolts and Si(100) were cleaned in an ultrasonic bath of detergent, alcohol, and deionized water for 30 min, respectively. Firstly, the AlN films were prepared at a growth temperature of 200 °C, target-to-bolts-distance of 100 mm, sputtering power of 3000 W, and Ar:N_2_ ratio of 30%. Secondly, a circular ring mask plate was covered on the AlN films to control the diameter of the top electrode. Thirdly, top electrode Mo films were deposited at a target-to-bolts-distance of 100 mm, sputtering power of 1500 W, and Ar flow of 70 sccm. The AlN thin film ultrasonic transducer is shown in Figure 1a. The outer Mo electrode is connected to bolts, and the effective area of the transducer depends on the diameter of the central Mo electrode.

The crystal structure of the AlN film was confirmed by X-ray diffraction (XRD, Ultima IV, Rigaku, Tokyo, Japan) with Cu *K*α radiation (λ = 0.154 nm) in the range of 30° ≤ 2θ ≤ 40° with a step of 0.01°. The surface morphology and cross-sectional microstructure of the AlN transducers were studied using a scanning electron microscope (SEM, Apreo 2C, Thermofisher, Waltham, MA, USA) operated at 10 kV. The piezoelectric constant *d*_33_ was measured using a quasi-static *d*_33_ tester (ZJ-3, IACAS, Beijing, China). The ultrasonic echo of the transducers was carried out by an ultrasonic pulser-receiver (USM-1, IFAST, Beijing, China) with a voltage of 60 V and a gain of 20 dB or 40 dB (shown in Figure 1b). The ultrasonic pulser-receiver generates an ultrashort electrical pulse signal, which is transmitted to the AlN transducer by a coaxial probe and converted into an ultrasonic wave signal through the piezoelectric effect. The ultrasonic wave propagates in the bolts, reflects from its bottom, and is converted back to the electrical signal by the same AlN transducer. The echo signal is further processed by the pulser-receiver and displayed on the computer. The values of peak-to-peak (Vpp) were used for evaluating the intensity of the ultrasonic echo.

The pretightening stress in GH4169 nickel base alloy bolts can be calculated by the change in time of flight (TOF) measured from the pulse-echo before and after tightening. The relationship between the TOF change and pretightening stress can be expressed as follows [10]:

(1) $$F=\frac{E\times S}{L}\times \frac{∆t\times v}{2}$$

where *F* is the pretightening stress, *E* is the elastic modulus of the bolt, *S* is the sectional area, *L* is the clamping length of the bolt, Δ*t* is the change in TOF before and after tightening, and *v* is the ultrasonic wave speed in the bolt. 

### 2.2. Theoretical Model

**Figure 2:**
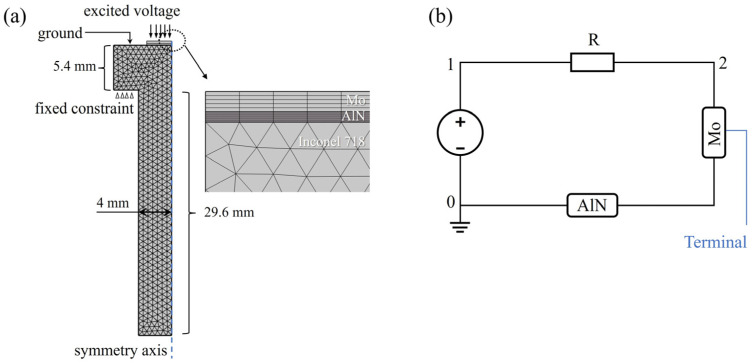
Two-dimensional axisymmetric theoretical model (**a**) and piezoelectric coupling circuit (**b**) of the AlN thin film transducer. The bolt structure is simplified, featuring no threads and approximate dimensions.

To reveal the enhancement mechanism of the ultrasonic echo in the AlN thin film transducer, the propagation process of ultrasound inside the nickel base alloy bolt was simulated by the finite element method. As shown in Figure 2a, the piezoelectric layer AlN and top electrode Mo are covered on the simple bolt model with an Inconel 718 nickel base alloy. The analytical function of the excited voltage on the top electrode is:

(2) $$f\left(t\right)=V_{0}\times \sin\left(2\times \pi \times f_{0}\times t\right)\times e^{{\left(-\frac{t-2\times T}{T}\right)}^{2}}$$

where *V*_0_ is the excitation voltage with the value of 60 V and *f*_0_ is the frequency with the value of 6 MHz, *T* is the period with the value of 1/*f*_0_. The ground is applied to the upper surface of the bolt head, while a fixed constraint is applied to the lower surface of the bolt head. Moreover, we can achieve more accurate results in our simulations by employing detailed mesh partitioning. A triangular mesh is utilized for the simple bolt model, while a mapping mesh is employed for both the electrode layer Mo and the piezoelectric layer AlN. The model has been optimized by changing the size of the Mo and AlN. In order to transmit and receive signals simultaneously, a reported piezoelectric coupling circuit is applied to the theoretical model [30] (shown in Figure 2b).

**Figure 3:**
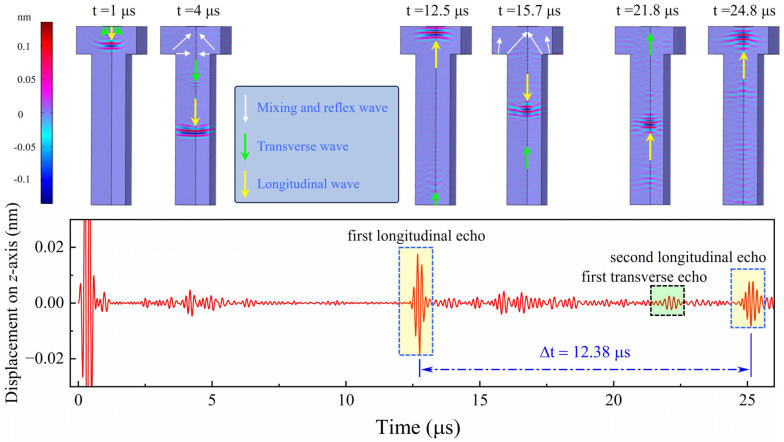
The propagation process of ultrasonic wave in the simple AlN thin film ultrasonic transducer model by finite element method.

The propagation process of the longitudinal wave, transverse wave, mixing and reflex wave on the bolt is shown in Figure 3. The longitudinal echo goes across the inside of the bolt twice and firstly reaches the bolt head at approximately 12.5 μs, while the second longitudinal echo reaches it at approximately 24.8 μs. We can calculate that the velocity of the longitudinal wave on the Inconel 718 bolt is about 5654 m/s. Mixing and reflex waves are observed at approximately 4 μs and 15.7 μs, and the first transverse echo is observed at approximately 21.8 μs, all of which can be detected by the AlN thin film ultrasonic transducer on the bolt head. In our work, we will place emphasis on the intensity of the first longitudinal echo of the ultrasound. Moreover, the displacement on the *z*-axis, which is derived from theoretical results, is regarded as the ultrasonic response.

## 3. Results and Discussion

### 3.1. Piezoelectric Layer Thickness Design

**Figure 4:**
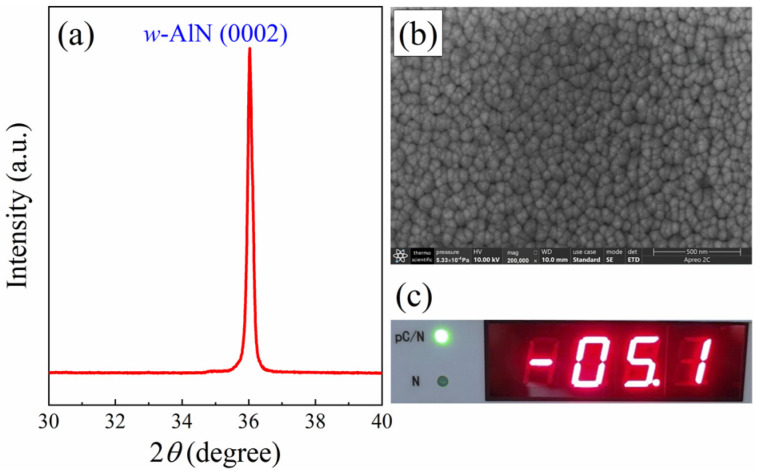
The XRD (**a**), SEM image (**b**) and *d*_33_ (**c**) of AlN thin film deposited by RF magnetron sputtering.

The thickness of the piezoelectric layer is a critical parameter in the design of ultrasonic transducers, as it significantly influences the transduction efficiency and overall performance of the device. In this section, we focus on the piezoelectric layer thickness design to enhance the intensity of the AlN thin film ultrasonic transducer. The AlN thin film was deposited by RF magnetron sputtering (shown in Figure 4). To facilitate the sample preparation, the samples of the XRD pattern and SEM image were deposited on Si(100). The XRD result shows the (0002) preferred orientation for the AlN thin film suggests that the film has a crystallographic texture on the *c*-axis. The SEM image clearly demonstrates a uniform distribution of fine grains on a dense surface. The *d*_33_ value, which is a measure of the piezoelectric coefficient, is given as 5.1 pC/N for the AlN thin film. This value is close to the reported value of 5.5 pC/N [15]. Overall, the combination of XRD, SEM, and *d*_33_ measurements provides a comprehensive assessment of the AlN thin film’s quality, indicating that it has been successfully prepared with desirable characteristics for its intended applications.

**Figure 5:**
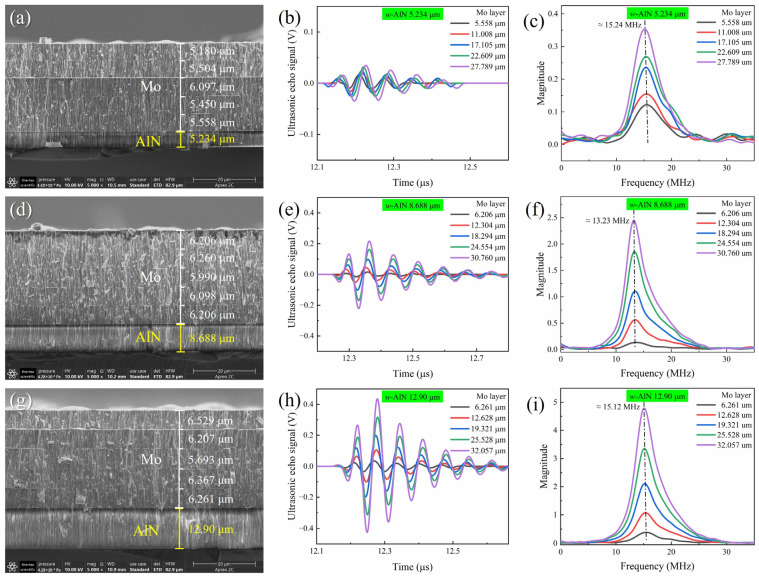
The cross-SEM image (**a**,**d**,**g**), ultrasonic signal (**b**,**e**,**h**), and frequency response (**c**,**f**,**i**) of AlN transducers with different thickness of AlN and Mo film layer deposited by RF magnetron sputtering.

**Figure 6:**
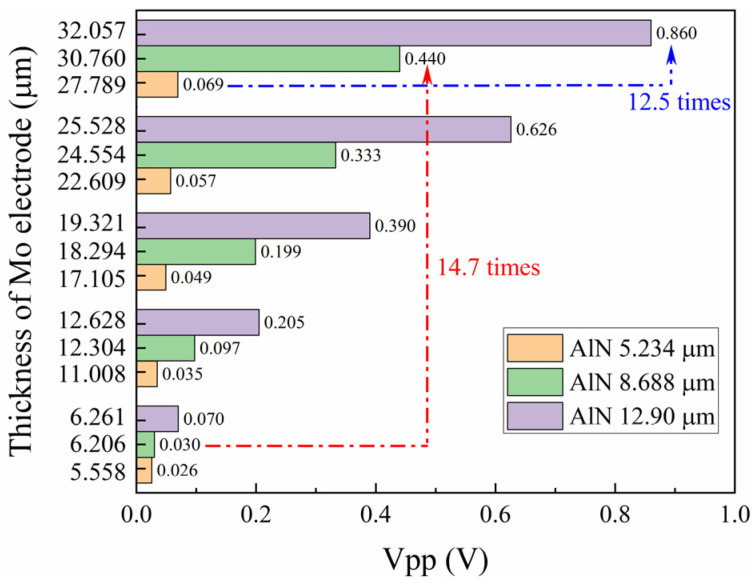
The Vpp varies with the thickness of Mo electrode for AlN thin film transducers.

Three piezoelectric AlN thin films, with thicknesses of 5.234 μm, 8.688 μm, and 12.90 μm, were deposited on GH4169 bolts and Si(100) by RF magnetron sputtering. Subsequently, five layers of Mo electrode thin films were deposited on the AlN thin film. Figure 5a,d,e show the cross-sectional microstructure of the three AlN thin films, which are aligned perpendicularly to the Si(100) substrate surface. This alignment is consistent with the *c*-axis orientation observed in the XRD results. The ultrasonic echo signals of the AlN thin film transducers, each with a different thickness AlN thin film and an approximately equal thickness Mo electrode, are plotted in Figure 5b,e,h. The applied gain of the ultrasonic pulser-receiver is set to 20 dB to ensure that the signal is amplified sufficiently for clear detection without causing distortion. The arrival times of the ultrasonic echo signals on the graph differ for the three thicknesses of the AlN thin films, primarily because of the slight variations in the length of the GH4169 bolts. The Vpp of the ultrasonic echo signals increases with the thickness of the AlN thin films, as shown in Figure 6. Specifically, the Vpp of the AlN thin film with a thickness of 12.90 μm is 12.5 times greater than that of the film with a thickness of 5.234 μm when the Mo electrode thickness is approximately 30 μm. The thickness increase of the AlN thin film piezoelectric layer typically results in a stronger signal output due to its enhanced ability to accumulate an electrical charge and produce a mechanical displacement [13,31]. Furthermore, the frequency response of the ultrasonic signals was obtained by the Fast Fourier Transform (FFT) method (shown in Figure 5c,f,i). It is well known that the resonant frequency decreases as the thickness of the piezoelectric increases. However, the center frequencies of transducers with AlN thin films with thicknesses of 5.234 μm, 8.688 μm, and 12.90 μm are 15.24 MHz, 13.23 MHz, and 15.12 MHz, respectively. The measured frequency is independent of the piezoelectric thickness. Another researcher has also found the same result in a LiNbO_3_ transducer for bolts [13]. Both of us think that this issue is caused by the non-resonant vibration modes within the bolt transducer. The AlN transducer utilizes pulse signals that typically encompass a wide frequency range, rather than being confined to a single resonant frequency.

**Figure 7:**
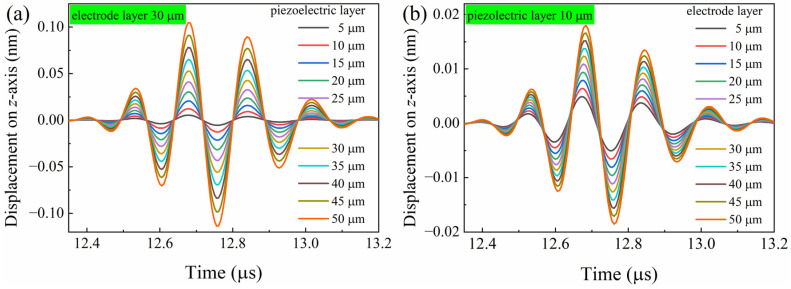
The simulated ultrasonic echo signals with different thickness of piezoelectric layer (**a**) and electrode layer (**b**) by finite element method.

In theory, the ultrasonic echo signals from AlN transducers with varying piezoelectric layer thicknesses were emulated using the finite element method, as shown in Figure 7a. Clearly, the intensity of the ultrasonic echo signal from the AlN transducer models increases with the thickness of the piezoelectric layer increasing, up to 50 μm. This result is consistent with the experimental results (shown in Figure 6) obtained under controlled laboratory conditions. The alignment between theoretical predictions and experimental data validates the accuracy of the finite element method in modeling the behavior of AlN transducers. Experimentally, if the phenomenon of AlN thin film layer shedding during the coating process is overlooked, the intensity of the ultrasonic echo signal is expected to increase with the layer thickness.

### 3.2. Electrode Layer Thickness Design

The electrode layer thickness is also a pivotal design parameter in the construction of ultrasonic transducers, as it directly influences the efficiency of the electrical-to-mechanical energy conversion processes. Five layers of Mo electrode thin films were successively deposited onto the AlN thin film using RF magnetron sputtering. Measurements of the AlN thin film transducer properties were conducted following the deposition of each Mo layer. Figure 5 presents the cross-sectional microstructure, ultrasonic echo signal, and frequency data for three samples, each with five layers of Mo thin films. As depicted in Figure 5a,d,g, each Mo layer in the five-layer structure is approximately 6 μm thick, contributing to a total thickness of 30 μm. The sharp and uncracked boundaries among the Mo layers in all samples indicate excellent adhesion between the layers, which minimizes the possibility of delamination and improves the mechanical robustness of the thin film structure. The ultrasonic echo signals of the AlN thin film transducers, each with five layers of a Mo electrode, are plotted in Figure 5b,e,h. The arrival times of ultrasonic echo signals on the graph are identical for a specific transducer. The intensity of ultrasonic echo signals increases with the thickness of the Mo thin films, and this trend is illustrated by the Vpp depicted in Figure 6. The Vpp of the Mo thin film with a thickness of 30 μm is significantly higher than that of the film with a thickness of 6 μm. This comparison is made with the AlN piezoelectric layer thickness at 8.688 μm, where the former’s Vpp is 14.7 times greater. The increasing trend is in agreement with the results of the theoretical simulation (shown in Figure 7b), with the electrode layer thickness ranging from 5 μm to 50 μm. Figure 5c,f,i also present the frequency response derived from ultrasonic signals for Mo films with varying thicknesses. Even though the electrode thickness varies, the frequency of ultrasonic echo signals basically remains unchanged when the transducer is the same.

**Figure 8:**
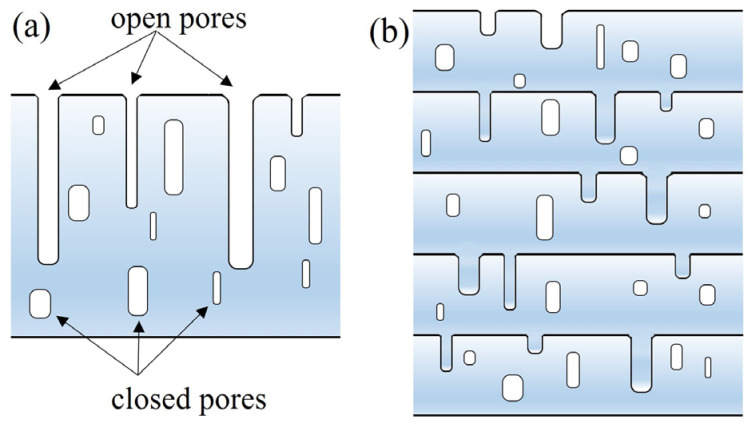
Schematic diagram of monolayer electrode structure (**a**) and multilayered electrode structure (**b**).

In physical vapor deposition techniques, it is difficult for deposited films to reach thicknesses of 30 μm or even 50 μm due to the presence of layer stress and intrinsic stress that can lead to delamination or cracking. In our work, the multilayered Mo electrode structure was successfully deposited layer-by-layer using RF magnetron sputtering, achieving a thickness of 30 μm without any delamination (shown in Figure 5a,d,g). Generally, the layer stress between layers of Mo is greater than that between Mo and AlN. Furthermore, the layer stress between Mo and AlN is greater than that between GH4169 and AlN. So, delamination will not occur in the multilayered Mo structure unless the intrinsic stresses of the multilayered structure are greater than the layer stress between Mo and AlN or between GH4169 and AlN. In addition, there are open pores and closed pores in the continuously deposited films [32], as shown in Figure 8a. Closed pores within the film cannot exchange water with the atmosphere during the heating or cooling processes. In contrast, open pores on the surface can exchange water with the atmosphere, and this exchange will be detrimental to the propagation of ultrasonic signals, potentially causing attenuation or distortion. The open pores on the surface can be filled in as subsequent layers are deposited using the layer-by-layer method, as shown in Figure 8b. The densification in a multilayered structure is expected to be greater than that in a single-layer film of equivalent thickness. This increased densification, coupled with strong interfacial bonding—a critical feature that ensures efficient electrical signal transfer across the multilayered electrode—significantly enhances the mechanical properties of the thin film. Consequently, the multilayered Mo structure can have a clear impact on the ultrasonic signals of a piezoelectric device.

### 3.3. Electrode Diameter Design

**Figure 9:**
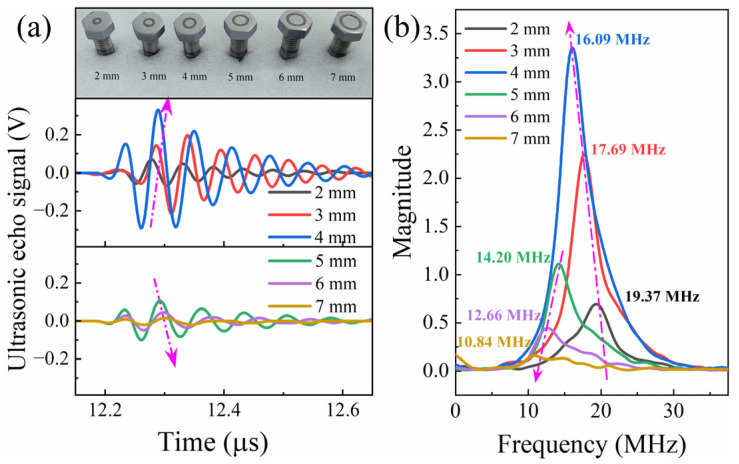
The ultrasonic echo signal (**a**) and frequency of ultrasonic signal (**b**) with different diameter of Mo film layer deposited by RF magnetron sputtering.

The diameter of the electrode in ultrasonic transducers determines the active area of the ultrasonic signals. Nevertheless, a larger diameter for the electrode is not necessarily better. The transducers with varying electrode diameters were deposited on GH4169 bolts by RF magnetron sputtering to explore the ultrasonic echo signals, as shown in Figure 9a. The applied gain of 40 dB was set in the ultrasonic pulser-receiver to optimize the signal-to-noise ratio. Observations show that the intensity of ultrasonic echo signals increases as the electrode diameter increases from 2 mm to 4 mm, but decreases when the diameter is further increased from 5 mm to 7 mm. Figure 9b gives the frequencies of the ultrasonic signal obtained using the FFT method. As the electrode diameter increases, the center frequency decreases and the waveform of the ultrasound signal becomes elongated. The reduction in the center frequency with larger electrode diameters can be attributed to the altered boundary conditions at the interface between the electrode and the piezoelectric layer, affecting the resonance characteristics of the transducer [33]. Generally speaking, lower-frequency waves tend to penetrate materials more deeply than higher-frequency waves, but the frequency magnitude and signal intensity decrease when the electrode diameter reaching exceeds 4 mm. Further study is needed on this aspect of simulation modeling.

**Figure 10:**
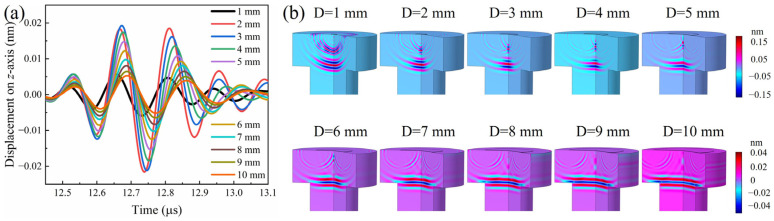
The simulated ultrasonic echo signal of AlN transducers with different diameters of electrode layers by finite element method (**a**), the ultrasonic signal with different diameters of electrode layers at 12.5 μs (**b**).

Figure 10a displays the simulated ultrasonic signals for AlN transducer models with electrodes of varying diameters ranging from 1 mm to 10 mm, which encompasses the range tested in the experiment. Similarly, the intensity of ultrasonic echo signals increases as the electrode diameter increases from 1 mm to 3 mm, but decreases when the diameter is further increased from 4 mm to 10 mm. The propagation of signals at 12.5 μs indicates that more and more signals are resisted and reflected at the interface between the bolt head and the screw when the diameter exceeds 4 mm (shown in Figure 10b). Consequently, the intensity of the ultrasonic echo signal decreases, even though the frequency decreases. The above results indicate that an electrode diameter of nearly 4 mm is optimal for an AlN thin film transducer of M8 bolts. Furthermore, the observed decrease in ultrasonic frequency with an increasing electrode diameter may impact the spatial resolution of imaging applications, necessitating a trade-off between sensitivity and resolution in the transducer design.

### 3.4. Pretightening Stress Measurement

**Figure 11:**
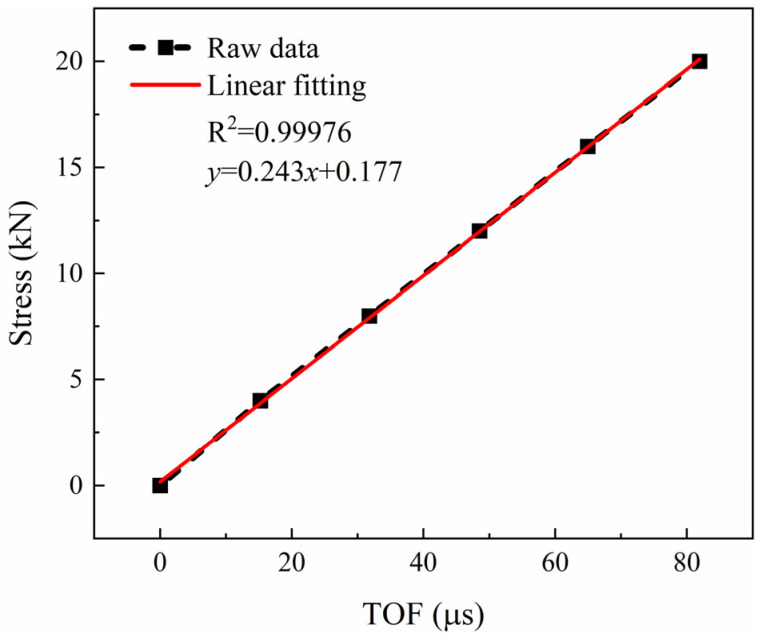
The calibration curve of AlN transducer sample, with an AlN layer of 12.90 μm and a Mo layer of 32.057 μm.

To further investigate the suitability of the AlN transducer for bolt applications, the bolts were load-tested at room temperature. The pretightening stress can be obtained by Equation (1) and the steps for measuring the pretightening stress of the bolt are as follows: Firstly, install the bolt on the tooling, test the echo signal of the unstressed state, and set the position of the echo signal to zero. Secondly, a known force equivalent to the pretightening stress is applied by the tension machine. Record the TOF between the echo signals obtained before pretightening and after setting to zero. Thirdly, plot the data on a graph to create a calibration curve that shows the relationship between the pretightening stress and the TOF. Finally, use the calibration curve to predict the pretightening stress. The AlN transducer sample, featuring an AlN layer measuring 12.90 μm and a Mo layer measuring 32.057 μm, and exhibiting the strongest intensity echo signal, was calibrated and the pretightening stress was measured. Figure 11 shows the calibration curve for a tension ranging from 0 to 20 kN. The determination coefficient R^2^ of the fitting line is 0.99976, confirming that the calibration process for measuring the pretightening stress is highly accurate. A series of stress tests was applied to the AlN transducer and the predicted stress was obtained by the calibration curve, as shown in Table 1. The percentage error was obtained from the predicted stress and actual stress. It was noted that the absolute error of this system was below ±0.2 kN, and the relative error was less than ±2.5%. This result is more accurate than those reported in other literature for both the PMTS [5] and other ultrasonic methods [30].

## 4. Conclusions

This research investigated the influence of the structure on the performance of AlN thin-film transducers, specifically in terms of ultrasonic echo signal intensity. Using RF magnetron sputtering, AlN film transducers were deposited on GH4169 nickel-based alloy bolts. The study meticulously examined the effects of varying the piezoelectric layer thickness, the electrode layer thickness, and the electrode diameter on the echo signal’s intensity. With the increasing thickness of the AlN piezoelectric layer, the maximum observed increase in echo signal intensity is 12.5 times greater than that of the thinnest layers. The thickness increase in the AlN thin film piezoelectric layer typically results in a stronger signal output due to its enhanced ability to accumulate an electrical charge and produce a mechanical displacement. With the increasing thickness of the Mo electrode layer, the maximum observed increase in echo signal intensity is 14.7 times greater than that of the thinnest layers. The multilayered Mo structure was employed to relieve inherent stress and ensure optimal adhesion between layers using the layer-by-layer method, which contributes to better densification. With the increasing diameter of the Mo electrode, the intensity of the echo signal first increases and then decreases due to the reflection at the interface between the bolt head and screw. An electrode diameter of nearly 4 mm is optimal for an AlN thin film transducer of M8 bolts. The pretightening stress is measured by an AlN thin film transducer, resulting in a lower error rate of ±2.5%. The study is significant for the field of non-destructive testing, particularly in optimizing transducer designs for applications such as measuring the pretightening stress in bolts. The optimal transducer structure proposed in this research is capable of receiving strong and stable echo signals, even under varying pretightening stress conditions.

## Figures and Tables

**Table 1:** The predicted pretightening stress results of AlN transducer sample, with an AlN layer of 12.90 μm and a Mo layer of 32.057 μm.

Actual Stress (kN)	Predicted Value (kN)	TOF (μs)	Percentage Error (%)
3	2.95	11.41	−1.67
6	5.85	23.33	−2.50
9	8.84	35.61	−1.78
12	11.94	48.36	−0.50
15	15.04	61.13	0.27
18	18.11	73.73	0.61
21	21.18	86.38	0.86

## Data Availability

Data are contained within the article.
